# CT synthesis in the head & neck region for PET/MR attenuation correction: an iterative multi-atlas approach

**DOI:** 10.1186/2197-7364-2-S1-A31

**Published:** 2015-05-18

**Authors:** Ninon Burgos, M Jorge Cardoso, Marc Modat, Shonit Punwani, David Atkinson, Simon R Arridge, Brian F Hutton, Sébastien Ourselin

**Affiliations:** Translational Imaging Group, Centre for Medical Image Computing, University College London, London, UK; Dementia Research Centre, University College London, London, UK; Division of Imaging, University College London Hospitals, London, UK; Centre for Medical Imaging, University College London, London, UK; Centre for Medical Image Computing, University College London, London, UK; Institute of Nuclear Medicine, University College London Hospitals, London, UK

In this work, we propose to tackle the problem of attenuation correction in the head and neck by synthesising CT from MR images using an iterative multi-atlas approach. The proposed method relies on pre-acquired T2-weighted MRI and CT images of the neck. For each subject, the MRI is non-rigidly mapped to the CT. To synthesise a pseudo CT, all the MRIs in the database are first registered to the target MRI. This registration consists of a robust affine followed by a non-rigid registration. The pseudo CT is obtained by fusing the mapped atlases according to their morphological similarity to the target. In contrast to CTs, T2 images do not provide a good estimate of the bone location. Combining multiple modalities at both the registration and image similarity stages is expected to provide more realistic mappings and to reduce the bias. An initial pseudo CT (pCT) is combined with the target MRI to form a MRI-pCT pair. The MRI-pCT pair is registered to all the MRI-CT pairs from the database. An improved pseudo CT is obtained by fusing the mapped MRI-CT pairs according to their morphological similarity to the target MRI-pCT pair. Results showed that the proposed CT synthesis algorithm based on a multi-atlas information propagation scheme and iterative process is able to synthesise pseudo CT images in a region challenging for registration algorithms. The results also demonstrate that the robust affine decreases the absolute error compared to the classic approach and that the bone refinement process reduces the bias in the bone region. The proposed method could be used to correct for attenuation PET/MR data, but also for dosimetry calculations in the context of MR-based radiotherapy treatment planning.

